# Professional quality of life among migrant and non-migrant mental health staff in Germany: a gender-sensitive cross-sectional study

**DOI:** 10.1038/s41598-026-63345-x

**Published:** 2026-07-28

**Authors:** Luisa Eilinghoff, Eric Hahn, Kerem Böge, Solveig Kemna, Malek Bajbouj, Van Phi Nguyen, Thi Minh Tam Ta

**Affiliations:** 1 Charité Universitätsmedizin, Charitéplatz 1, 10117 Berlin, Germany; 2 Freie Universität Berlin, Berlin, Germany; 3 Deutsches Zentrum für psychische Gesundheit, Berlin, Germany; 4 Medizinische Hochschule Brandenburg Theodor Fontane, Neuruppin, Germany; 5 Hanoi Medical University, Hanoi, Vietnam

**Keywords:** Professional Quality of Life, Migration, Burnout, Gender, Mental Health Workforce, Germany, Health care, Health humanities, Health occupations, Psychology, Psychology, Risk factors

## Abstract

Germany increasingly relies on an internationally recruited migrant workforce, like Vietnamese migrants, to address shortages in the mental healthcare sector. This cross-sectional study examined differences in the Professional Quality of Life (ProQOL), measured through the subscales burnout (BO), secondary traumatic stress (STS) and compassion satisfaction (CS), among Vietnamese migrant and non-migrant mental health professionals (*N* = 312) in Germany. Participants completed an online survey in German or Vietnamese including the ProQOL – 5 Scale, as well as sociodemographic and work-related variables. Multiple linear regression models identified salary across all subscales and psychiatric pre-conditions for BO and STS as significant predictors for ProQOL outcomes. Analyses of covariance compared adjusted group means across migration and gender. Vietnamese migrant professionals report significantly lower CS and higher STS compared to non-migrant professionals, while no significant migration-related differences were observed for BO. Considering gender, women exhibit significantly higher CS than men, whereas no significant gender differences emerged for BO or STS. No significant migration × gender interaction effects were found. Findings indicate migration background is associated with relevant differences in ProQOL among mental health staff in Germany, underscoring the need for targeted support, like an inclusive workplace culture and work-related social network. Further research should incorporate direct measures of migration-related stressors to better understand underlying mechanisms and longitudinal designs.

## Introduction

### Vietnamese migrants and the mental health workforce in Germany

Germany represents the second-largest host country of international migrants worldwide, with 29.7% of its population having a migration background^1^. This includes approximately 208,000 Vietnamese migrants, who represent the largest Southeast Asian migrant group^1^. The Vietnamese population in Germany is characterized by a distinctive and well-documented migration history since the 1950s, which differs from that of other migrant groups. It began with the state-organized migration of students and contract workers to the German Democratic Republic (GDR) in the 1950s, followed by refugee movements in the 1970s during the Vietnam War, and from the 1990s onwards, there was economically motivated migration for education and work. These heterogeneous waves of migration have led to established community structures and differentiated educational and professional integration pathways^2,3^.

In parallel migrants are crucial in meeting the demand for healthcare professionals^4^, the proportion rose from 16.9% in 2013 to 22% in 2019, while the number of foreign professionals more than doubled during that period^5^. In mental healthcare, the workforce shortage is especially acute: In 2023, no psychiatric hospital in Germany met the minimum staffing requirements, with only 56% of wards adequately staffed with nurses and 50% with social workers, while 17% lacked psychotherapists and 27% were understaffed with psychiatrists. As a reason, 75% of psychiatric hospitals stated that the vacancies could not be filled due to a shortage of specialists^6^. In response, Germany intensified international recruitment from non-EU countries through bilateral programs (e.g. “Triple Win”) including partnerships with Vietnam, which illustrates Germany’s approach to training and integrating foreign professionals^7–9^. Vietnamese migrants make a notable contribution to Germany’s workforce, particularly in the healthcare sector^7,8^. The combination of the historical roots of migration described above, institutionalized migration channels, and integration into the labor market makes Vietnamese professionals a distinct and historically identifiable subgroup within the German healthcare workforce. In parallel, the reliance on migrant healthcare professionals highlights their importance but also raises concerns about the psychosocial burden and occupational stress.

### Migration-related stress and mental health risks

Migrants face numerous stressors across all migration phases: *pre-migration stressors*, like political violence, crime, trauma and poverty, *stressors during migration*, including family separation, the loss of personal belongings and *post-migration stressors*, such as legal uncertainties, language barriers, non-recognition of qualifications, discrimination, lack of social networks and feelings of alienation^10–12^. Post-migration stressors can be described as acculturative stress - the psychological strain resulting from navigating the often-conflicting values, beliefs, and practices of the heritage and host countries. Such stress can lead to feelings of isolation, identity conflicts, and psychological strain^13^. Migration-related stress factors are associated with mental health disorders^10,12,14^, with 20% of migrants in Germany experiencing depressive symptoms^15^. Importantly, migration related stressors must be conceptually distinguished from the occupation-specific emotional demands inherent to the mental healthcare profession, which will be introduced in the next section.

### Job demand – resource model in mental healthcare

To conceptually integrate migration-related factors and occupational emotional demands in the workplace, we draw on the Job Demands–Resources Model (JD–R Model ; Demerouti et al., 2000). The model is based on the assumption that employees well-being decreases, and the risk of burnout increases when the demands of the job cannot be met through resources^16^. Demands require sustained effort, and involve costs (e.g. time pressure); while resources (e.g. collegial support) can serve to support the achievement of goals and generally reduce demands^16^.

Within the JD–R framework, migration-related stressors may be conceptualized as additional contextual demands^17^ that can be distinguished from, yet co-occur with, occupation-specific emotional demands inherent to mental healthcare work (e.g. acute crisis), that can accumulate the effort to meet professional expectations, particularly when access to resources (e.g. social support) is limited. Gender may influence exposure to demands and access to resources within this framework^18^.

In this framework, “Professional Quality of Life” (ProQOL; Stamm, 2005), which will be discussed below, can be seen as an outcome of the JD-R model in the field of care professions, while the JD-R model can be seen as an overarching framework.

### ProQOL framework

In mental healthcare professions, demands and resources are particularly influenced by the ongoing exposure to chronic psychiatric illness, severe trauma and acute crisis. To capture these profession-specific processes, the concept “Cost of Care” was introduced by Charles Figley (1995), which explores the emotional burdens experienced by caregivers such as nurses, social workers, psychologists, psychotherapists, and psychiatrists. This concept describes that while the care work is fulfilling, it inherently requires significant emotional labor, which can lead to compassion fatigue (CF), a state of reduced empathic capacity, divided into two parts: burnout (BO) and secondary traumatic stress (STS). While BO results from chronic workplace stress, STS arises from indirect exposure to patients’ trauma, leading to PTSD-like symptoms^19–22^. Conversely, compassion satisfaction (CS) refers to the emotional reward derived from helping, which can counterbalance the emotional demands and thereby reinforce professional motivation. Building on these concepts Figley (1995) and Stamm (2002), Stamm (2005) the ProQOL measurement was developed, which this study applies.

### Demands and resources associated with ProQOL outcome

A number of profession-specific demands were identified as contributing to a lower ProQOL. Exposure to direct or indirect violence and insufficient training in mental health-related fields exacerbate STS and BO^23–25^, while prior personal trauma increases the risk for STS^24–26^.

Beyond profession-specific demands, general occupational stressors such as job insecurity, interpersonal tensions, and excessive working hours have been associated with increased BO and STS^23,27,28^, while working overtime contributes to BO^27,28^.

Protective factors for ProQOL, which can also be categorized as profession-specific resources, include a strong professional identity, alignment with caregiving values, a healthy lifestyle (fitness, sleep routine), higher education/sufficient training and perceived social support as a buffer against the emotional toll, all of which correlate with higher CS and lower BO/STS^28–32^.

A general occupational resource is high income/satisfaction with the income, replicated by multiple studies^28,31,33^.

These ProQOL outcomes have direct implications for clinical care: high CS enhances motivation and a deeper understanding of patient needs^34^. Conversely, BO and STS are linked with emotional exhaustion, detachment from patients, committing medical errors and reduced care quality^35,36^. Importantly these profession-specific and general occupational demands differ from migration-related stressors in mental healthcare field, which will be examined next.

### ProQOL of migrant professionals

Migration related stressors that impact the ProQOL are low social support, high migratory grief and loss, and the absence of family in the new homeland all associated with higher BO^37^, while discrimination and racism is associated with overall lower ProQOL^27,38,39^. These stressors may contribute to dissatisfaction and staff attrition, including re-migration^40^.

Although ProQOL has already been extensively studied in mental health care settings, the influence of migration on professionals with a migration background in Germany remains largely unexplored.

ProQOL is particularly well suited for this study, as it captures stress-related and reward-related dimensions of professional well-being inherent to professional cargiving. Unlike more general occupational stress models, ProQOL differentiates between genereal occupational strain (e.g. time pressure) and profession-specific stress (e.g. acute crisis, exposure to trauma), which are especially salient in mental healthcare contexts^20,41,42^ and may be compounded by migration related stressors^24–26^.

Conceptually, these dimensions can be incorporated within the broader JD-R model, while maintaining a professional-specific operationalization tailored to mental health care.

### Gendered view of care work

Gender should not be understood merely as a socio demographic variable, but as a socially structured system of role expectations shaping behavior and occupational opportunities^43,44^. According to *Social Role Theory*^43^, women are expected to fulfill „communal roles“ characterized by e.g. selflessness, care, and emotional responsiveness. In contrast, men are expected to fulfill „agentic roles“ involving e.g. leadership or physical strength^43^. Care professions are structurally characterized by emotional labor and widely understood as a feminized field of work in which competencies, such as empathy, are required and socially associated with femininity^45–47^. Those gender role dynamics remain empirically visible until today: women constitute 67 per cent of the global care^48^. Nevertheless, it is discussed, that women in care professions may experience social recognition through consistency with societal gender expectations^44,49^. Consequently have higher job satisfaction than men^50^. In contrast, men working in female-dominated settings may experience role incongruence or backlash for engaging in “communal” work, which can result in reduced social legitimacy^51,52^, potentially limiting their experience of emotional reward derived from care work^53,54^.

To return to the ProQOL, higher CS in women was found in multiple studies^55–58^. Simultaneously, higher levels of BO^59,60^ and STS have been found among women^61^ with mixed findings^62^, suggesting that, while experiencing higher CS, women may simultaneously be more exposed to emotional demands and therefore experience higher BO and STS.

### Potential additive and intersectional effects of migration and gender on ProQOL outcomes

Intersectionality refers to overlapping and interconnected social categories that shape the opportunities and experiences of a person. It was originally developed to explain oppression based on gender and ethnicity^63^. Applied to the context of mental health professions, migration-related stressors, such as discrimination or limited social support^27,38,39,64^ may co-occur with gender-specific role expectations in care professions described above^45,47,51,52^. Whether these two dimensions complement one another additively or interact with one another – that is, whether professional experience has a multiplicative effect, as an intersectional perspective would predict – remains an open empirical question, which we will explore by examining their interaction.

To our knowledge, no study has jointly examined migration and gender in relation to ProQOL outcomes. Both factors may contribute independently, that is, additively, to ProQOL, while an intersectional perspective additionally raises the possibility that they interact, such that migration-related contextual demands do not affect women and men equally. We therefore examine the effects of migration and gender on ProQOL and test whether these factors combine additively or interactively, thereby addressing this research gap.

Overall, ProQOL in the care profession can be understood as the result of additive and/or overlapping demands and resources. Emotional labor, exposure to trauma, and chronic stress represent job-specific demands that can exacerbate BO and STS, while professional identity, social support, and adequate working conditions act as protective resources that promote CS. Migration-related stressors can represent additional demands that add to workplace stress. At the same time, gender-specific role expectations shape experiences at work and access to professional resources.

Taken together, migration and gender may influence ProQOL by altering the balance between demands and resources in mental health care settings, either as independent (additive) contributions or as an interactive (intersectional) pattern, a distinction we examine empirically.

Vietnamese mental health professionals in Germany represent a historically and institutionally relevant subgroup in which these dynamics can be examined.

### Study objectives and hypothesis

This study investigates how the ProQOL of mental health professionals differs depending on migration and gender and whether gender and migration interact in shaping ProQOL. Additionally, we exploratorily examine associations between selected occupational and clinical factors (e.g. salary, working hours, psychiatric pre-conditions) and the three subscales.

We expect (1) professionals with a Vietnamese migration background to report lower CS and higher BO and STS than non-migrant professionals. Additionally, we assume that (2) female caregivers, regardless of migration, report higher CS, but also higher BO and STS than males. Furthermore, we expect (3) a migration × gender interaction effect, such that female professionals with a migration background will report lower CS and higher BO and STS compared to female professionals without a migration background.

## Methods

### Procedure

The study was publicly announced October 2021 via SosciSurvey in German and Vietnamese through various professional channels, including mailing lists of psychiatric and psychosocial services, nursing and psychotherapeutic training institutes, health organizations, posts on hospital intranets, and social media groups for psychologists, nurses, and social workers. Several professional networks were approached. Vietnamese professional networks and community organizations were also included to ensure adequate representation of professionals with a Vietnamese migration background. Participation was open to all eligible mental health professionals, regardless of their migration background. Identical inclusion and exclusion criteria applied to all participants. Participants were included if they worked as mental health professionals (e.g., social workers, psychologists, psychotherapists, psychiatrists, nurses, etc.). Exclusion criteria were being a voluntary carer, retired, unemployed, or not holding a work permit. Of the 317 participants recruited, 5 had to be excluded, resulting in a total sample of 312 participants. Questionnaires were available as both online survey and paper-and-pencil formats. Participants received monetary compensation for completing the 30-minute task.

This study was approved by the Ethics Committee of Charité – Universitätsmedizin Berlin (reference number: EA1/133/21) in May 2021. All participants provided informed consent prior to participation. All methods were performed in accordance with the relevant guidelines and regulations, including the Declaration of Helsinki.

### Measures

The ProQOL is a widely used measure of the impact of caring for psychiatric or traumatised patients^42^. According to the terms of use provided by the ProQOL Office (Center for Victims of Torture), the ProQOL-5 may be freely copied and used for research purposes (www.proqol.org) provided that the source is credited and the measure is not sold.

**Fig. 1:**
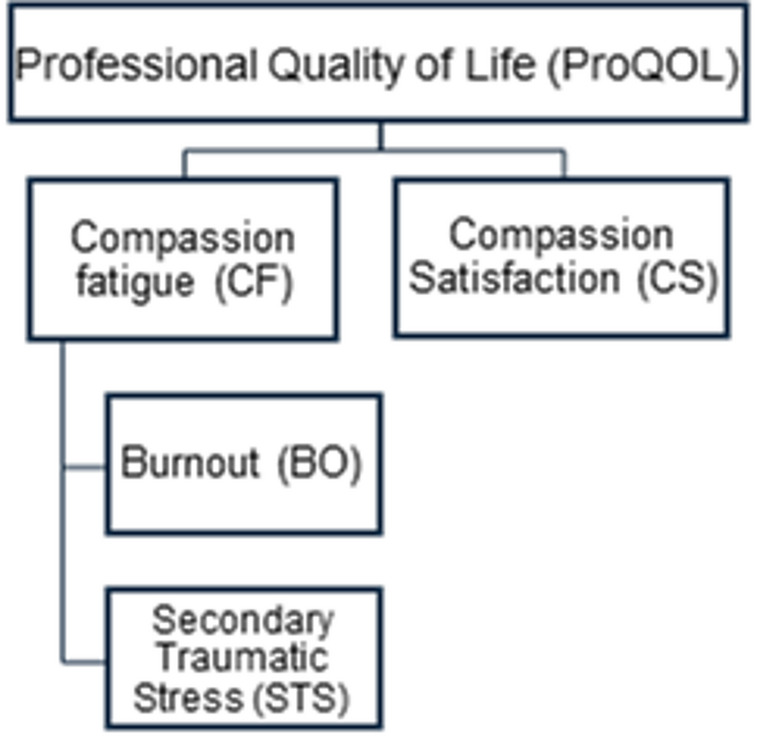
Structure of the ProQOL.

The ProQOL is defined as “the quality one person feels about their work as a helper”^42^. It includes three subscales: CS measures the emotional reward, such as experiencing meaning, fulfilment and societal contribution. In contrast, the burdensome aspects are compromised in the CF scale: BO summarizing feelings of emotional exhaustion, hopelessness linked to an excessive workload and/or a non-supportive environment with symptoms of fatigue, anger, rage, and depression; as well as the STS subscale, which results from exposure to trauma experienced by others and includes symptoms like insomnia, anxiety and intrusive thoughts (see Fig. 1).

ProQOL is used in over 26 languages^65^ and was validated in Vietnamese in 2023^28^, published after our data collection began. Therefore, we applied a translation-back-translation technique recommended by the WHO^66^ with a team of Vietnamese native speakers. Following the WHO guidelines, the translation process included forward translation by a bilingual native speaker, independent back-translation by a second translator, and reconciliation of discrepancies to ensure semantic equivalence. Our translation is congruent in 20 of 30 questions with the Vietnamese validated translation, while 10 questions differ in nuanced wording and temporal framing (e.g. “trauma” vs. “stressful event”)^28^. An example of this is Item 2, which shifts the focus from concern for many^28^ to cognitive preoccupation with one person (in our version). The instrument has demonstrated good reliability and validity across various studies, as evidenced in over 200 published papers^65^. In previous research, the validity of the subscales differs slightly: CS and STS have a strong internal consistency with a Cronbach’s alpha of > 0.80, while the BO subscale has a comparatively lower Cronbach’s alpha ranging from 0.60 − 0.70^67,68^. As shown in Table 1, Cronbach’s alpha was calculated separately for each subscale and each language. Previous results of Cronbach’s Alpha were replicated, with a comparatively low Cronbach’s alpha for BO (0.69 for German and 0.70 for the Vietnamese version), while > 0.80, for CS and STS^28,67,68^. Although the BO subscale has shown lower internal consistency in several studies, it represents a core dimension of the ProQOL framework and captures aspects of occupational stress that are conceptually distinct from STS^42^ and was therefore retained to ensure the conceptual completeness. The ProQOL utilises a 5-point Likert scale ranging from 1 (never) to 5 (always). A high total score in CS indicates a high level of compassion satisfaction, which is the desired expression. A high score on the BO and STS scale reflects a high level of symptoms associated with burnout or secondary traumatic stress. Overall, elevated scores for CS suggest a healthy professional quality of life, whereas BO and STS scores should ideally be as low as possible^42^.

**Table 1:** Cronbach’s Alpha values for the subscales.

Subscale	Language	Cronbach’s Alpha
CS	German	0.86
	Vietnamese	0.86
CF		
BO	German	0.69
	Vietnamese	0.70
STS	German	0.82
	Vietnamese	0.80

The Cronbach’s alpha values replicate values from recent studies using the ProQOL in German^67,68^ and Vietnamese^28^.

### Statistical analysis

Data were imported into SPSS (Version 27.0) and subsequently analysed, with significance set at *p* ≤ .05. After screening the data for errors, outliers, respondent misconduct, exclusion criteria and missing data, socio-eco-demographic variables were described using frequency, percentage, mean, and standard deviation. Sum scores for all subscales were calculated.

Multiple linear regression analyses (MLR) were conducted to explore significant associations between ProQOL subscales and individual and occupational characteristics previously reported in the literature as relevant correlates of the ProQOL^69,70^. Assumptions of MLR were examined prior to analysis, including linearity, homoscedasticity, normal distribution of residuals, and multicollinearity diagnostics. All covariates were entered simultaneously into MLR (enter method).

Two-way analysis of covariance (ANCOVA) was performed to test for group differences in ProQOL subscales by gender and migration as fixed factors, including a gender x migration interaction term. Salary (for all scales) and psychiatric pre-conditions (for STS and BO) were included as covariates based on prior empirical evidence identifying socioeconomic and mental health factors as potential confounders. Educational attainment was additionally included to adjust for structural differences in our sample. Prior to conducting the ANCOVAs assumptions including normality of residuals, homogeneity of variances, homogeneity of regression slopes, linearity, and absence of influential outliers, were examined prior to analysis and were not violated.

Group differences were evaluated using adjusted estimated marginal means derived from the ANCOVA models.

## Results

### Sample characteristics

Detailed socio-eco-demographic characteristics are summarized in Table 2. The 312 participants are divided into 151 non-migrant and 161 migrant caregivers. The overall sample was predominantly female (70.1% female vs. 29.9% male). Higher academic qualifications (master’s degree or higher) are more common among non-migrant caregivers (50.2%) than among migrants (9.3%). Additionally, 19.8% of the qualifications of migrant professionals are not recognised in Germany. The occupational status indicates that more migrant professionals are in training (38.4%) than non-migrant (11.9%) and more commonly full-time employed (76.9%), than non-migrant (59.4%). Non-migrant professionals prefer part-time work (38.9%), more than migrants (19.2%).

The group differences in educational attainment, occupational status, and recognition of qualifications may reflect structural disparities between migrant and non-migrant professionals and were therefore considered in the multivariable analyses by including education and salary as covariates.

### Multiple linear regression

Assumptions for MLR were tested prior to analysis. No substantial violations were detected. One standardized residual exceeding ± 3 SD was identified for CS and two for STS; these cases were retained given the large sample size and the robustness to isolated outliers (Berry, 1993).

The R² for the overall Model 1 (CS) was 0.095 (adjusted R² = 0.048), indicating low explained variance. This was higher for Model 2 (BO), where R² was 0.202 (adjusted R² = 0.161), and for Model 3 (STS), R² was 0.162 (adjusted R² = 0.119). Salary significantly predicts all subscales (*p* < .001), while psychiatric pre-existing medical conditions significantly predict STS and BO (*p* < .001) (see in Table 3**)**.

### Two-way factorial ANCOVA: group differences by migration, gender and interaction analysis

Assumptions for ANCOVA were examined prior to analysis. All assumptions were fullfilled, expect for BO, a small but statistically significant interaction between migration status and salary emerged (*p* = .048, partial η² = 0.009). Given the small effect size and the robustness of ANCOVA in large samples, results were interpreted with appropriate caution and this is listed as a limitation.

A two-way factorial ANCOVA was conducted for each ProQOL subscale, including migration status and gender as fixed factors. Salary^28,31,33^ and highest educational attainment^25,29,31^ were entered as covariates in all models based on prior empirical findings and observed sample differences in educational distribution. Psychiatric pre-conditions^20,21,24^ were additionally controlled for in the BO and STS models.

As shown in Table 4, a significant main effect of migration status emerged for CS (ηp² = 0.031) and STS (ηp² = 0.095). The effect size for migration status was small for CS and moderate for STS. No significant main effect was observed for BO. A significant main effect of gender was found for CS with a small effect size (ηp² = 0.020). No significant migration × gender interactions were observed for CS, BO, or STS.

Among covariates, salary was significantly associated with CS (ηp² = 0.023), BO (ηp² = 0.063) and STS (ηp² = 0.016). Psychiatric pre-conditions were significantly associated with BO (ηp² = 0.077) and STS (ηp² = 0.043), while educational attainment showed a significant association with BO only, (ηp² = 0.018).

### Exploratory gender-stratified migration effects

Gender-stratified adjusted means are presented in Table 5 for exploratory descriptive purposes, given the absence of significant migration × gender interactions. These adjusted marginal means indicated that caregivers with a migration background reported lower CS and higher STS scores compared to non-migrant professionals, whereas no migration-related differences were observed for BO.

**Table 2:** Socio-eco-demographic characteristics.

Characteristic	Non-migrant		Migrant	
	*n*	%*	*n*	%*
Total	151	100	161	100
Gender				
Male	52	34.4	45	26.9
Female	101	68.6	114	71.3
Language used in the study				
German	151	100	47	29.1
Vietnamese	0		114	70.7
Education in Germany				
Training/Specialised degree	43	28.4	79	49
Bachelor’s degree	25	16.5	25	15.5
Master’s degree or above	76	50.2	11	6.8
Other or no educational qualification (in Germany)	7	4.6	46	28.5
Education in Vietnam				
Associate degree after 2–3 yrs of Training^1^			17	10.5
Bachelor’s Degree^2^			21	13
Master’s degree or higher^3^			4	2.48
Other/no educational qualification			56	34.7
Acknowledgement of qualifications from VN				
Yes			28	17.4
No			32	19.8
Not necessary			38	23.6
Occupation				
Student	25	16.5	27	16.7
In training	18	11.8	63	39.1
Work time				
Full-time	90	59.4	124	76.9
Part-time	59	38.9	31	19.2

*Rounded to one decimal point; ^1^Bằng cao đẳng, ^2^Bằng Cử nhân đại học, ^3^Bằng thạc sỹ.

**Table 3:** Multiple regression model for factors impacting the CS, STS and BO subscales of the ProQOL.

Subscale ProQOL	Predictor	B	SE	ß	t	*p*	95% CI	
							LL	UL
CS	Age	0.023	0.041	0.037	0.559	0.577	− 0.057	0.103
	Gender	1.383	0.775	0.104	1.784	0.075	− 0.142	2.909
Work area	Clinical setting	− 0.259	0.863	− 0.021	− 0.300	0.764	− 1.958	1.439
	Integration Assistant	− 0.311	1.485	− 0.012	− 0.209	0.834	− 3.235	2.613
	Youth Services	− 1.208	1.220	− 0.063	− 0.990	0.322	− 3.610	1.193
	Migrations Assistant	− 0.867	1.341	− 0.039	− 0.646	0.518	− 3.506	1.772
	Salary	− 1.297	0.365	− 0.223	− 3.552	< 0.001	− 2.016	− 0.578
Status	Employed	− 0.111	0.800	− 0.009	− 0.139	0.888	− 1.687	1.463
	Self-employed	− 0.736	1.556	− 0.028	− 0.473	0.636	− 3.800	2.326
Work time	Full-time	− 1.417	1.586	− 0.107	− 0.893	0.372	− 4.539	1.704
	Part-time	0.514	1.643	0.037	0.312	0.754	− 2.721	3.749
Health	Psychiatric pre-conditions	− 1.570	0.936	− 0.096	− 1.676	0.094	− 3.414	0.273
	Physical pre-conditions	1.526	1.018	0.086	1.498	0.135	− 0.478	3.530
	Family Status	− 0.314	0.304	− 0.063	− 1.032	0.302	− 0.912	0.284
BO	Age	− 0.042	0.031	− 0.086	− 1.375	0.170	− 0.102	0.018
	Gender	− 0.239	0.579	− 0.023	− 0.414	0.679	− 1.379	0.9
Work area	Clinical setting	0.176	0.644	0.017	0.273	0.785	− 1.092	1.444
	Integration Assistant	0.202	1.109	0.010	0.182	0.856	− 1.981	2.385
	Youth Services	1.415	0.911	0.093	1.554	0.121	− 0.378	3.207
	Migrations Assistant	− 1.760	1.001	− 0.099	− 1.759	0.080	− 3.730	0.210
	Salary	1.376	0.273	0.297	5.045	< 0.001	0.839	1.912
Status	Employed	− 0.078	0.598	− 0.008	− 0.130	0.897	− 1.254	1.098
	Self-employed	0.530	1.162	0.026	0.456	0.649	− 1.757	2.816
Work time	Full-time	2.227	1.184	0.211	1.881	0.061	− 0.103	4.557
	Part-time	0.616	1.227	0.057	0.502	0.616	− 1.799	3.030
Health	Psychiatric pre-conditions	3.697	0.699	0.283	5.286	< 0.001	2.320	5.073
	Physical pre-conditions	− 0.892	0.760	− 0.063	− 1.174	0.242	− 2.389	0.604
	Family Status	0.075	0.227	0.019	0.331	0.741	− 0.372	0.521
STS	Age	− 0.054	0.039	− 0.089	− 1.390	0.165	− 0.131	0.022
	Gender	1.107	0.741	0.084	1.494	0.136	− 0.351	2.565
Work area	Clinical setting	− 0.303	0.824	− 0.024	− 0.367	0.713	− 1.927	1.319
	Integration Assistant	0.325	1.420	0.012	0.229	0.818	− 2.469	3.120
	Youth Services	1.906	1.166	0.101	1.634	0.103	− 0.389	4.201
	Migrations Assistant	− 1.909	1.281	− 0.086	− 1.490	0.137	− 4.432	0.612
	Salary	1.111	0.349	0.193	3.184	0.001	0.424	1.798
Status	Employed	− 1.170	0.765	− 0.095	− 1.529	0.127	− 2.676	0.335
	Self-employed	1.701	1.487	0.067	1.143	0.253	− 1.225	4.629
Work time	Full-time	2.709	1.516	0.206	1.786	0.074	− 0.274	5.692
	Part-time	1.133	1.570	0.084	0.721	0.471	− 1.958	4.225
Health	Psychiatric pre-conditions	3.638	0.895	0.225	4.063	< 0.001	1.876	5.400
	Physical pre-conditions	− 1.292	0.973	− 0.074	− 1.327	0.185	− 3.207	0.623
	Family Status	0.750	0.227	0.019	0.331	0.741	− 2.389	0.604

**Table 4:** Two-way ANCOVA examining effects of migration and gender on ProQOL subscales.

Dependent variable (Subscale)	Independent variable	F (df)	*p*	ηp^2^
CS^a^	Migration	9.536 (1)	0.002	0.031
	Gender	6.297 (1)	0.013	0.020
	Migration x Gender	0.110 (1)	0.740	0.000
	Salary (Covariate)	7.018 (1)	0.008	0.023
	Education (Covariate)	0.173	0.678	0.001
CF				
BO^b^	Migration	2.464 (1)	0.118	0.008
	Gender	1.058 (1)	0.304	0.004
	Migration x Gender	0.319 (1)	0.572	0.001
	Salary (Covariate)	20.550 (1)	< 0.001	0.064
	Psychiatric Pre-conditions (Covariate)	25.015 (1)	< 0.001	0.077
	Education (Covariate)	5.598 (1)	0.019	0.018
STS^b^	Migration	31.401 (1)	< 0.001	0.095
	Gender	2.077 (1)	0.151	0.007
	Migration x Gender	0.966	0.326	0.003
	Salary (Covariate)	4.959 (1)	0.027	0.016
	Psychiatric Pre-conditions (Covariate)	13.579 (1)	< 0.001	0.043
	Education (Covariate)	0.002 (1)	0.964	0.000

^a^Salary and education level as a covariate.

^b^Salary, education level and psychiatric pre-conditions as covariates. ηp² = partial eta squared.

**Table 5:** Adjusted means and exploratory gender-stratified migration effects for ProQOL subscales.

Subscale	Gender	Migration	M adj	SE	Migration Effect F(df)	*p*	ηp²
CS^a^	Women	No	38.705	0.621			
		Yes	35.884	0.583	10.102(1)	0.002	0.032
	Men	No	36.628	0.877			
		Yes	34.292	0.897	3.256(1)	0.072	0.011
CF							
BO^b^	Women	No	25.425	0.465			
		Yes	26.104	0.436	0.106(1)	0.745	0.000
	Men	No	25.678	0.659			
		Yes	26.977	0.675	1.267(1)	0.261	0.004
STS^b^	Women	No	21.223	0.576			
		Yes	24.622	0.541	2.940(1)	0.087	0.010
	Men	No	19.576	0.817			
		Yes	21.223	0.836	0.103(1)	0.749	0.000

M adj = adjusted mean based on gender-stratified ANCOVAs; SE = standard error; ηp² = partial eta squared. ^a^Salary as a covariate ^b^salary and psychiatric pre-conditions as covariates. These exploratory analyses were conducted despite non-significant migration × gender interactions in the primary models (Table 4).

## Discussion

The results confirmed assumption (1) partly: migrant professionals exhibit significantly lower CS and higher STS, whereas no significant migration-related differences were observed for BO.

Assumption (2) was also partially confirmed: while women experience significantly higher CS than men, no significant gender differences were found for BO and STS. No significant migration × gender interactions were observed across any subscale, indicating that gender did not moderate migration-related differences. Assumption (3) that migrant female carers do have the lowest CS, while the highest BO and STS was not supported, due to the nonsignificant gender x migration interactions. Exploratory stratified analyses suggested descriptively more pronounced migration-related differences among women, though these effects did not differ significantly by gender.

Among covariates, salary was significantly associated with all subscales^24,31,33^ and psychiatric pre-conditions with BO and STS^24,25^. Educational attainment was additionally included as a covariate to account for structural differences between migrant and non-migrant professionals that may confound migration-related group comparisons.

### Compassion satisfaction

Across all groups, CS values were moderate^42^ and predicted by salary^24,31,33^. Migrant professionals reported significantly lower CS. Lower CS may be consistent with migration-related factors discussed in literature, such as employment insecurity, language barriers, unrecognised qualifications^11^, discrimination^38^, and lower social support^28,71^, which may limit access to the intrinsic reward of caregiving roles. For example, 19.8% of foreign qualifications were not recognized in the present sample, which may indicate structural barriers to full professional integration. Prior studies suggest, that insufficient training, limited career progression or underemployment are associated with lower CS^29,31,33^ and limiting career advancement^72^.

Further, social support has been identified as a protective factor for CS^28,37,73^, but migrants may experience reduced access because family and friends are not living in Germany^74^. However, as migrations-related stressors were not directly assessed, these interpretations remain hypothetical and should be examined more explicitly in future research.

Gender differences showed significantly higher CS scores in women than in men, regardless of migration, consistent with previous studies^56–58^.

This pattern may be consistent with societal recognition arising from gender-role congruence in care professions, which could support women’s intrinsic reward^49,51,75^. Conversely, lower CS among men might reflect perceived incongruence with gender roles (backlash phenomena), which could distance them from societal validation^45,47,52–54,76^ or be associated with social penalisation for working in a female-dominated profession^51^. In line with the “Social Role Theory”, women are expected to embody “communal” traits such as care, emotional availability and empathy^43,49^, an alignment that may be consistent with higher CS.

Because mechanisms such as perceived role congruence or social recognition were not directly assessed in the present study, these interpretations remain tentative and require further research. With a small effect size, salary (ηp² = 0.023) was associated with CS, in line with prior studies linking financial security and perceived professional reward to higher levels of compassion satisfaction^24,31,33^. However, as salary was included as a theoretically informed covariate rather than a primary predictor, this association should be interpreted as exploratory.

### Burnout

BO levels were moderate across all groups. In contrast to CS and STS, no significant migration-related differences in BO were observed after adjusting for salary, educational attainment, and psychiatric pre-conditions.

This absence of group differences may partly reflect measurement-related limitations, as the internal consistency of the German BO subscale in the present sample was relatively low (German scale Cronbach’s α = 0.69; Vietnamese scale Cronbach’s α = 0.70), potentially attenuating detectable group effects. In addition, adjustment for educational attainment reduced previously observed differences, suggesting that structural disparities between migrant and non-migrant professionals, such as differences in formal qualifications, may contribute to BO levels independent of migration status per se, consistent with prior findings^25,29,31^.

Among covariates, psychiatric pre-conditions and salary were significantly associated with BO in the adjusted model, which replicates findings from prior studies. Among covariates, both psychiatric pre-conditions (ηp² = 0.077) and salary (ηp² = 0.063) showed moderate associations with BO, suggesting that individual clinical and structural factors may play a more prominent role in BO than migration status per se^24,25,77^. These effects were not subject to a priori hypotheses and are therefore interpreted as exploratory.

### Secondary traumatic stress

STS levels for non-migrant professionals were significantly lower than those for their migrant counterparts, who experienced moderate STS^42^ with a moderate effect size (ηp² ≈ 0.095). This difference remained after adjusting for salary, educational attainment, and psychiatric pre-conditions.

Although psychiatric pre-conditions were associated with STS in the adjusted models^24,25^, participants were not explicitly asked about trauma history. STS may be associated with traumatic events of the caregivers lifetime^24,77^. It is conceivable, that migration-related traumatic experiences, which do not have pathological value and are therefore not captured as “psychiatric pre-conditions”, may account for the higher STS scores observed among migrants^77^. In this study, it cannot be determined whether migration-related experiences contributed to higher STS levels, as trauma history was not directly assessed. Future research would benefit from examining migration-related stressors or trauma exposure more explicitly to clarify the mechanisms underlying STS differences.

Further, no significant gender differences were found for STS, in line with mixed findings of systematic reviews^62^.

### Migration and gender interaction

In all ProQOL subscales, no statistically significant interaction between migration and gender was observed in the adjusted ANCOVA models. Taken together, these results indicate that, in the present sample, migration and gender were associated with ProQOL in an additive rather than an intersectional (multiplicative) manner. The intersectional framework thus served as a heuristic that motivated the test of the interaction, rather than as a model confirmed by the data. The absence of significant interaction effects suggests that migration-related differences did not statistically vary by gender in the present sample. Gender-adjusted means were retained for exploratory descriptive purposes: descriptively lower CS among migrant women compared to non-migrant women were found, whereas differences among men were smaller in magnitude. These findings should be interpreted as hypothesis-generating for further research, given the absence of a statistically significant interaction effect. Furthermore, the unequal gender distribution in the migration groups may have limited the statistical power to detect potential interaction effects, as explained in the limitations section.

### Integration from the perspective of the JD-R model

From the perspective of the JD-R model^16^, the present findings reflect differences in the balance between contextual demands and available professional resources^16,78,79^. Although migration-related factors are not directly assessed, they may represent additional demands that exist alongside the emotional demands of working in mental health care^17^. The lower CS and higher STS observed among migrants may be tentatively interpreted as consistent with a less favourable demand–resource constellation, although these constructs were not directly assessed. Gender differences in CS can be interpreted within this framework, as different expectations of social roles may influence access to and perception of resources^43,79^. However, as these processes were not measured directly, such interpretations remain tentative. Consistent with an additive rather than an intersectional account, the absence of significant interactions between migration and gender indicates that, in this sample, migration- and gender-related influences were associated with ProQOL independently rather than synergistically. Overall, the results suggest that a distinction should be made between the different dimensions of ProQOL, as CS, BO and STS may respond differently to demands and resources.

### Policy and practical implications

The observed migration-related differences in CS and STS, as well as gender differences in CS, highlight the importance of structural support for healthcare professionals. While the present study does not directly assess specific migration-related stressors, prior literature suggests fast track recognition of qualifications, as recommended by the UN and the Expert Council of Integration and Migration^11,80,81^ accompanied by modular occupation-specific advanced training programs, when formal equivalence of qualifications cannot be granted^11^would promote workforce integration, satisfaction and reduce attrition rates^11,80^. Additionally, establishing support networks, such as mentorship programs, may enhance workplace adaptation^82,83^, provide emotional and professional guidance, and increase CS while reducing BO^28,73,83,84^. To address the burden of caregiving, psychiatric institutions may provide access to resilience-focused interventions, including mindfulness-based programs, psychoeducation workshops, and coping strategy training. Those interventions can contribute to workforce wellbeing and satisfaction^42,83,85–87^, while fostering retention and quality of care and therefore contributing to the sustainability of the German healthcare system, which is increasingly reliant on internationally recruited staff^11^.

### Limitations

This cross-sectional study provides important insights into the ProQOL of Vietnamese migrants and German mental healthcare professionals, but limitations must be noted. First, its cross-sectional design precludes causal inferences^88^, and the reliance on self-reported data introduces the possibility of response biases^89^. Secondly, although the total sample size was sufficient, the gender distribution across migration groups was uneven, resulting in relatively small subgroup sizes particularly among males. Unequal cell sizes can reduce the statistical power for detecting interactions and increase the instability of parameter estimates in smaller subgroups^90^.Consequently, gender effects and interactions between migration and gender (not significant in this case) must be interpreted with caution.

Third, a violation of the assumption of homogeneity of regression slopes was observed in the BO subscale. Furthermore, the comparatively low internal consistency of the BO scale (α = 0.69/0.70) may have weakened the effect estimates; therefore, the BO-related results should be interpreted with caution.

Fourth, the explained variance for CS was low (adjusted R² = 0.048), indicating limited predictive power of the variables included in the MLR. A high proportion of migrant professionals were in training or entry-level positions, which affects generalisability^91^.

Last, neither migration-related stressors (such as discrimination and recognition of qualifications) nor the gender-related mechanisms invoked in the discussion (such as perceived role congruence, trauma and social recognition) were directly assessed; therefore, interpretations regarding migration- and gender-related contextual factors and their underlying mechanisms remain tentative and should be examined more directly in future research.

### Future research

Future research should employ longitudinal approaches to capture changes in ProQOL and to better understand potential causal pathways. To enable a more accurate assessment of the potential interactions between migration and gender, larger samples with more balanced subgroup sizes are required. In addition, psychometric evaluation of the BO subscale in different linguistic contexts is still necessary. Next, studies with more balanced subgroup sizes are needed to enable a more accurate estimation of potential interactions.

Future research should incorporate additional contextual and structural factors to better capture the broader professional environment in which migrant professionals work.

Including more experienced professionals in both groups would improve generalizability, while assessing workplace metrics or mixed methods approaches could enhance robustness. Cross-national comparative studies across healthcare systems and migrant populations could further clarify structural influences in migration related determinants.

## Conclusion

This study examined migration- and gender-related differences in the ProQOL of mental health professionals in Germany. Professionals with a Vietnamese migration background reported lower CS and higher STS, while no significant migration-related differences were found for BO. Women reported higher CS than men. No significant migration × gender interactions were found, indicating that the two factors were associated with ProQOL in an additive rather than an intersectional manner. These findings suggest that migration status and gender are relevant, and apparently independent, contextual factors for understanding the dimensions of ProQOL in mental health care. Further research is needed to clarify the underlying mechanisms and identify contextual and structural conditions that can promote professional well-being in an increasingly international mental health workforce.

## Data Availability

The datasets used and/or analysed during the current study are available from the corresponding author on reasonable request.

## Abbreviations

ANCOVA: Analysis of covariance
BO: Burnout
CF: Compassion fatigue
CI: Confidence interval
CS: Compassion satisfaction
EU: European Union
GDR: German Democratic Republic
GIZ: Deutsche Gesellschaft für Internationale Zusammenarbeit (German Agency for International Cooperation)
JD-R: Job Demands-Resources
MDiff: Mean difference
MLR: Multiple linear regression
ProQOL: Professional Quality of Life
PTSD: Post-traumatic stress disorder
SE: Standard error
SD: Standard Deviation
SPSS: Statistical Package for the Social Sciences
STS: Secondary traumatic stress
UN: United Nations
WHO: World Health Organization

## Code availability

No custom code, bespoke software, or new algorithm was generated for this study. All statistical analyses were performed using IBM SPSS Statistics (version 27; IBM Corp., Armonk, NY, USA). As no custom code was used, there is no code associated with this manuscript to deposit.

## References

[1] Statitisches Bundesamt. *Bevölkerung in Privathaushalten 2023 nach Migrationshintergrund*. (2023). https://www.destatis.de/DE/Themen/Gesellschaft-Umwelt/Bevoelkerung/Migration-Integration/Tabellen/migrationshintergrund-staatsangehoerigkeit-staaten.html;jsessionid=97B9597B1B67076CD03512BF51CB858F.internet8721

[2] Bösch, F. & Zentrum Für Zeithistorische Forschung. Engagement für Flüchtlinge: Die Aufnahme vietnamesischer „Boat People in der Bundesrepublik. *Zeithistorische Forschungen*. 10.14765/ZZF.DOK.4.760 (2017).

[3] Schmiz, A. *Transnationalität als Ressource? Netzwerke vietnamesischer Migrantinnen und Migranten zwischen Berlin und Vietnam* (1. Aufl.). transcript Verlag. (2011). 10.14361/transcript.9783839417652

[4] Smith, J. B., Herinek, D., Woodward-Kron, R. & Ewers, M. Nurse Migration in Australia, Germany, and the UK: A Rapid Evidence Assessment of Empirical Research Involving Migrant Nurses. *Policy Politics Nurs. Pract.* **23** (3), 175–194. 10.1177/15271544221102964 (2022). 10.1177/15271544221102964 PMC9274786 35747915

[5] Expert Council on Integration und Migration. *Systemrelevant: Der Beitrag von Zugewanderten im Gesund- heitswesen*. (2022). https://www.svr-migration.de/wp-content/uploads/2022/10/SVR_Factsheet_Jahresgutachten_2022.pdf

[6] Deutsches Krankenhausinstitut. *PSYCHIATRIE BAROMETER 2023/2024*. (2024). https://www.dkgev.de/fileadmin/default/Mediapool/1_DKG/1.7_Presse/1.7.1_Pressemitteilungen/2024/2024-07-12_PM_Anlage_DKI-Psych-Barometer.pdf

[7] GIZ. *Expertise for Viet Nam, Care Staff for Germany*. (2017). https://reporting.giz.de/2017/our-work-around-the-world/displacement-and-migration/expertise-for-viet-nam-care-staff-for-germany/index.html

[8] GIZ. *Recruiting Trainees from Viet Nam*. (2020). https://www.giz.de/en/worldwide/80962.html

[9] WHO. *WHO and partners call for urgent investment in nurses*. (2020). https://www.who.int/news-room/detail/07-04-2020-who-and-partners-call-for-urgent-investment-in-nurses

[10] Anisman, H., Doubad, D., Asokumar, A. & Matheson, K. Psychosocial and neurobiological aspects of the worldwide refugee crisis: From vulnerability to resilience. *Neurosci. Biobehavioral Reviews*. **165**, 105859. 10.1016/j.neubiorev.2024.105859 (2024). 10.1016/j.neubiorev.2024.105859 39159733

[11] Expert Council on Integration und Migration. *A Crucial Component. Migration – Support and Challenge for Germany’s Healthcare System- Annual Report 2022*. (2022). https://www.svr-migration.de/wp-content/uploads/2022/05/SVR-Annual-Report-2022_Core-Messages.pdf

[12] Foo, S. Q. et al. Prevalence of Depression among Migrants: A Systematic Review and Meta-Analysis. *International Journal of Environmental Research and Public Health*, *15*(9), 1986. (2018). 10.3390/ijerph15091986 10.3390/ijerph15091986 PMC6163821 30213071

[13] Berry, W. D. *Understanding regression assumptions* (Sage, 1993).

[14] Jannesari, S., Hatch, S., Prina, M. & Oram, S. Post-migration Social–Environmental Factors Associated with Mental Health Problems Among Asylum Seekers: A Systematic Review. *J. Immigr. Minor. Health*. **22** (5), 1055–1064. 10.1007/s10903-020-01025-2 (2020). 32430778 10.1007/s10903-020-01025-2 PMC7441054

[15] Bartig, S. *Health of people with selected citizenships: Results of the study GEDA Fokus*. (2023). 10.25646/11143 10.25646/11143 PMC10091045 37064418

[16] Demerouti, E., Bakker, A. B., Nachreiner, F. & Schaufeli, W. B. The job demands-resources model of burnout. *J. Appl. Psychol.* **86** (3), 499–512. 10.1037/0021-9010.86.3.499 (2001). 11419809

[17] Qin, X., Hom, P., Xu, M. & Ju, D. Applying the job demands–resources model to migrant workers: Exploring how and when geographical distance increases quit propensity. *J. Occup. Organizational Psychol.* **87** (2), 303–328. 10.1111/joop.12047 (2014).

[18] Xie, X., Huang, C., Cheung, S. P., Zhou, Y. & Fang, J. Job Demands and Resources, Burnout, and Psychological Distress of Social Workers in China: Moderation Effects of Gender and Age. *Front. Psychol.* **12**, 741563. 10.3389/fpsyg.2021.741563 (2021). 34955962 10.3389/fpsyg.2021.741563 PMC8702995

[19] Bride, B. E., Radey, M. & Figley, C. R. Measuring Compassion Fatigue. *Clin. Soc. Work. J.* **35** (3), 155–163. 10.1007/s10615-007-0091-7 (2007).

[20] Figley, C. R. *Compassion fatigue: Coping with secondary traumatic stress disorder in those who treat the traumatized.* Brunner/Mazel. (1995). https://www.researchgate.net/publication/285028560_Secondary_traumatic_stress_disorder_An_overview

[21] Figley, C. R. Compassion fatigue: Psychotherapists’ chronic lack of self care. *J. Clin. Psychol.* **58** (11), 1433–1441. 10.1002/jclp.10090 (2002). 12412153 10.1002/jclp.10090

[22] Pehlivan, T. Compassion Fatigue: The Known, Unknown. *J. Psychiatric Nurs.*10.14744/phd.2017.25582 (2017).

[23] Adeyemo, S. O. et al. Experiences of Violence, Compassion Fatigue and Compassion Satisfaction on the Professional Quality of Life of Mental Health Professionals at a Tertiary Psychiatric Facility in Nigeria. *Sci. J. Clin. Med.* **3**, 6969 (2015).

[24] Hamid, A., Scior, K., Abdul-Hamid, W. & Williams, A. C. D. C. Displaced Syrian Mental Health Workers: An Investigation of Professional Quality of Life. *J. Refugee Stud.* **34** (2), 2394–2405. 10.1093/jrs/feaa068 (2021).

[25] Sijbrandij, M., Acarturk, C., Bird, M., Bryant, R. A., Burchert, S., Carswell, K.,De Jong, J., Dinesen, C., Dawson, K. S., El Chammay, R., Van Ittersum, L., Jordans,M., Knaevelsrud, C., McDaid, D., Miller, K., Morina, N., Park, A.-L., Roberts, B.,Van Son, Y., … Cuijpers, P. (2017). Strengthening mental health care systems for Syrian refugees in Europe and the Middle East: Integrating scalable psychological interventions in eight countries. European Journal of Psychotraumatology, 8(sup2), 1388102. https://doi.org/10.1080/20008198.2017.1388102. 10.1080/20008198.2017.1388102 PMC5687806 29163867

[26] Henderson, C. et al. Mental health-related stigma in health care and mental health-care settings. *Lancet Psychiatry*. **1** (6), 467–482. 10.1016/S2215-0366(14)00023-6 (2014). 26361202 10.1016/S2215-0366(14)00023-6

[27] Shell, E. M., Teodorescu, D. & Williams, L. D. Investigating Race-related Stress, Burnout, and Secondary Traumatic Stress for Black Mental Health Therapists. *J. Black Psychol.* **47** (8), 669–694. 10.1177/00957984211033963 (2021).

[28] Tran, A. N. P., To, Q. G., Huynh, V. A. N., Le, K. M. & To, K. G. Professional quality of life and its associated factors among Vietnamese doctors and nurses. *BMC Health Serv. Res.* **23** (1), 924. 10.1186/s12913-023-09908-4 (2023). 37649084 10.1186/s12913-023-09908-4 PMC10469419

[29] Başoğul, C., Arabaci, B., Mutlu, L., Satıl, E. & Büyükbayram Aslan, A. Professional values and professional quality of life among mental health nurses: A cross-sectional study. *Nurs. Health Sci.* **23** (2), 362–371. 10.1111/nhs.12811 (2021). 33433046 10.1111/nhs.12811

[30] Geoffrion, S., Lamothe, J., Giguère, C. É. & Collin-Vézina, D. The effects of adherence to professional identity, workplace aggression, and felt accountability on child protection workers’ professional quality of life. *Child Abuse Negl.* **135**, 105950. 10.1016/j.chiabu.2022.105950 (2023). 36410288 10.1016/j.chiabu.2022.105950

[31] Hamaideh, S. et al. Professional Quality of Life, Job Satisfaction, and Intention to Leave among Psychiatric Nurses: A Cross-Sectional Study. *Nurs. Rep.* **14** (2), 719–732. 10.3390/nursrep14020055 (2024). 38651467 10.3390/nursrep14020055 PMC11036228

[32] Magliano, L. et al. The Influence of Causal Explanations and Diagnostic Labeling on Medical Studentsʼ Views of Schizophrenia. *Acad. Med.* **86** (9), 1155–1162. 10.1097/ACM.0b013e318226708e (2011). 21785312 10.1097/ACM.0b013e318226708e

[33] Mangoulia, P., Koukia, E., Alevizopoulos, G., Fildissis, G. & Katostaras, T. Prevalence of Secondary Traumatic Stress Among Psychiatric Nurses in Greece. *Arch. Psychiatr. Nurs.* **29** (5), 333–338. 10.1016/j.apnu.2015.06.001 (2015). 26397438 10.1016/j.apnu.2015.06.001

[34] Kulkarni, S., Bell, H., Hartman, J. L. & Herman-Smith, R. L. Exploring Individual and Organizational Factors Contributing to Compassion Satisfaction, Secondary Traumatic Stress, and Burnout in Domestic Violence Service Providers. *J. Soc. Social Work Res.* **4** (2), 114–130. 10.5243/jsswr.2013.8 (2013).

[35] Klein, J. & von dem Knesebeck, O. Inequalities in health care utilization among migrants and non-migrants in Germany: A systematic review. *Int. J. Equity Health*. **17** (1), 160. 10.1186/s12939-018-0876-z (2018). 30382861 10.1186/s12939-018-0876-z PMC6211605

[36] Pergol-Metko, P., Staniszewska, A., Metko, S., Sienkiewicz, Z. & Czyzewski, L. Compassion Fatigue and Perceived Social Support among Polish Nurses. *Healthcare* **11** (5), 706. 10.3390/healthcare11050706 (2023). 36900712 10.3390/healthcare11050706 PMC10001227

[37] Farrell, I. C., Basma, D., DeDiego, A. C., Maurya, R. K. & Hurt-Avila, K. M. Predictors of burnout for immigrant mental health professionals in the United States. *Int. J. Social Welf.* **33** (1), 178–187. 10.1111/ijsw.12595 (2024).

[38] DeDiego, A. C., Farrell, I. C., Basma, D. & Maurya, R. K. Impact of immigration and discrimination on vocational wellness for immigrant mental health professionals. *J. Employ. Couns.* **61** (2), 118–135. 10.1002/joec.12222 (2024).

[39] Shell, E. M., Hua, J. & Sullivan, P. Cultural racism and burnout among Black mental health therapists. *J. Employ. Couns.* **59** (3), 102–110. 10.1002/joec.12187 (2022).

[40] Pantenburg, B., Kitze, K., Luppa, M., König, H. H. & Riedel-Heller, S. G. Physician emigration from Germany: Insights from a survey in Saxony, Germany. *BMC Health Serv. Res.* **18** (1), 341. 10.1186/s12913-018-3142-6 (2018). 29743052 10.1186/s12913-018-3142-6 PMC5944134

[41] Stamm, B. Measuring compassion satisfaction as well as fatigue: Developmental history of the Compassion Satisfaction and Fatigue Test. *Treating compassion fatigue*. (2002).

[42] Stamm, B. H. *The Concise ProQOL Manual: The concise manual for the Professional Quality of Life Scale* (2. Aufl.). (2010). https://www.researchgate.net/publication/340033923_The_Concise_ProQOL_Manual_The_concise_manual_for_the_Professional_Quality_of_Life_Scale_2_nd_Edition

[43] Eagly, A. H. *Sex Differences in Social Behavior: A Social-role interpretation* (1. Aufl.). Psychology Press. (1987). 10.4324/9780203781906

[44] Ridgeway, C. L. *Framed by Gender: How Gender Inequality Persists in the Modern World* (Oxford University Press, 2011). 10.1093/acprof:oso/9780199755776.001.0001

[45] England, P. Emerging Theories of Care Work. *Ann. Rev. Sociol.* **31** (1), 381–399. 10.1146/annurev.soc.31.041304.122317 (2005).

[46] Hochschild, A. R. *The Managed Heart: Commercialization of Human Feeling*. (1983). https://www.jstor.org/stable/10.1525/j.ctt1pn9bk

[47] England, P. The Gender Revolution: Uneven and Stalled. *Gend. Soc.* **24** (2), 149–166. 10.1177/0891243210361475 (2010).

[48] Limani, D. & Sodergren, M. C. *Where women work: Female-dominated occupations and sectors*. International Labour Organization. (2023). https://ilostat.ilo.org/where-women-work-female-dominated-occupations-and-sectors/#:~:text=Unsurprisingly%2C%20women%20still%20occupy%20traditionally,as%20those%20related%20to%20apparel

[49] Eagly, A. H. & Karau, S. J. Role congruity theory of prejudice toward female leaders. *Psychol. Rev.* **109** (3), 573–598. 10.1037/0033-295X.109.3.573 (2002). 12088246 10.1037/0033-295x.109.3.573

[50] Kuo, T. S., Chu, L. C., Shih, C. L., Li, Y. C. & Kao, P. L. Emotional labor, job satisfaction, and retention among home care workers in Taiwan: A comprehensive analysis. *Front. Psychol.* **16**, 1545955. 10.3389/fpsyg.2025.1545955 (2025). 40231001 10.3389/fpsyg.2025.1545955 PMC11994665

[51] Heilman, M. E. & Wallen, A. S. Wimpy and undeserving of respect: Penalties for men’s gender-inconsistent success. *J. Exp. Soc. Psychol.* **46** (4), 664–667. 10.1016/j.jesp.2010.01.008 (2010).

[52] Rudman, L. A. & Fairchild, K. Reactions to Counterstereotypic Behavior: The Role of Backlash in Cultural Stereotype Maintenance. *J. Personal. Soc. Psychol.* **87** (2), 157–176. 10.1037/0022-3514.87.2.157 (2004). 10.1037/0022-3514.87.2.157 15301625

[53] MacWilliams, B. R., Schmidt, B. & Bleich, M. R. Men in Nursing. *AJN Am. J. Nurs.* **113** (1), 38–44. 10.1097/01.NAJ.0000425746.83731.16 (2013). 23247678 10.1097/01.NAJ.0000425746.83731.16

[54] Stanley, D. et al. The male of the species: A profile of men in nursing. *J. Adv. Nurs.* **72** (5), 1155–1168. 10.1111/jan.12905 (2016). 26799533 10.1111/jan.12905

[55] O’Sullivan, J. & Whelan, T. A. Adversarial growth in telephone counsellors: Psychological and environmental influences. *Br. J. Guidance Couns.* **39** (4), 307–323. 10.1080/03069885.2011.567326 (2011).

[56] Padmanabhanunni, A. The cost of caring: Secondary traumatic stress and burnout among lay trauma counsellors in the Western Cape Province. *South. Afr. J. Psychol.* **50** (3), 385–394. 10.1177/0081246319892898 (2020).

[57] Prost, S. G. & Middleton, J. S. Professional quality of life and intent to leave the workforce: Gender disparities in child welfare. *Child Abuse Negl.* **110**, 104535. 10.1016/j.chiabu.2020.104535 (2020). 32448643 10.1016/j.chiabu.2020.104535

[58] Salloum, A., Kondrat, D. C., Johnco, C. & Olson, K. R. The role of self-care on compassion satisfaction, burnout and secondary trauma among child welfare workers. *Child Youth Serv. Rev.* **49**, 54–61. 10.1016/j.childyouth.2014.12.023 (2015).

[59] Greenglass, E. R., Burke, R. J. & Ondrack, M. A Gender-role Perspective of Coping and Burnout. *Appl. Psychol.* **39** (1), 5–27. 10.1111/j.1464-0597.1990.tb01035.x (1990).

[60] Sprang, G., Clark, J. J. & Whitt-Woosley, A. Compassion Fatigue, Compassion Satisfaction, and Burnout: Factors Impacting a Professional’s Quality of Life. *J. Loss Trauma.* **12** (3), 259–280. 10.1080/15325020701238093 (2007).

[61] Baum, N. Secondary Traumatization in Mental Health Professionals: A Systematic Review of Gender Findings. *Trauma. Violence Abuse*. **17** (2), 221–235. 10.1177/1524838015584357 (2016). 25964278 10.1177/1524838015584357

[62] Fernández, S., Guiote, J. M. & Miró, E. Review of Protective and Predisposing Factors in the Vicarious Traumatization of Psychotherapists. *Papeles Del. Psicólogo - Psychol. Papers*. **45** (2), 65–72. 10.23923/pap.psicol.3034 (2024).

[63] Crenshaw, K. *Mapping the Margins: Intersectionality, Identity Politics, and Violence against Women of Color*. (1991). https://www.jstor.org/stable/1229039?seq=1&cid=pdf-reference#references_tab_contents

[64] Berry, J. W. Acculturation and Adaptation in a New Society. *Int. Migration*. **30** (s1), 69–85. 10.1111/j.1468-2435.1992.tb00776.x (1992).

[65] Stamm, B. H. *Comprehensive Bibliography of Documents Specifically Using the ProQOL Measure.* www.proqol.org (2016).

[66] WHO (Hrsg.). (2012). *WHODAS 2.0 Translation Package (Version 1.0) Translation and linguistic evaluation protovol and supporting material*. https://terrance.who.int/mediacentre/data/WHODAS/Guidelines/WHODAS%202.0%20Translation%20guidelines.pdf

[67] Heritage, B., Rees, C. S. & Hegney, D. G. The ProQOL-21: A revised version of the Professional Quality of Life (ProQOL) scale based on Rasch analysis. *PLOS ONE*. **13** (2), e0193478. 10.1371/journal.pone.0193478 (2018). 29489875 10.1371/journal.pone.0193478 PMC5831102

[68] Galiana, L., Arena, F., Oliver, A., Sansó, N. & Benito, E. Compassion Satisfaction, Compassion Fatigue, and Burnout in Spain and Brazil: ProQOL Validation and Cross-cultural Diagnosis. *J. Pain Symptom Manag.* **53** (3), 598–604. 10.1016/j.jpainsymman.2016.09.014 (2017). 10.1016/j.jpainsymman.2016.09.014 28062348

[69] McGrath, K., Matthews, L. R. & Heard, R. Predictors of compassion satisfaction and compassion fatigue in health care workers providing health and rehabilitation services in rural and remote locations: A scoping review. *Aust. J. Rural Health*. **30** (2), 264–280. 10.1111/ajr.12857 (2022). 35267227 10.1111/ajr.12857 PMC9310831

[70] Unjai, S., Forster, E. M., Mitchell, A. E. & Creedy, D. K. Predictors of compassion satisfaction among healthcare professionals working in intensive care units: A cross-sectional study. *Intensive Crit. Care Nurs.* **79**, 103509. 10.1016/j.iccn.2023.103509 (2023). 37541068 10.1016/j.iccn.2023.103509

[71] Xie, W. et al. Prevalence and factors of compassion fatigue among Chinese psychiatric nurses: A cross-sectional study. *Medicine* **99** (29), e21083. 10.1097/MD.0000000000021083 (2020). 32702852 10.1097/MD.0000000000021083 PMC7373503

[72] Salami, B., Meherali, S. & Covell, C. L. Downward occupational mobility of baccalaureate-prepared, internationally educated nurses to licensed practical nurses. *Int. Nurs. Rev.* **65** (2), 173–181. 10.1111/inr.12400 (2018). 28786097 10.1111/inr.12400

[73] Xie, W. et al. The prevalence of compassion satisfaction and compassion fatigue among nurses: A systematic review and meta-analysis. *Int. J. Nurs. Stud.* **120**, 103973. 10.1016/j.ijnurstu.2021.103973 (2021). 34102372 10.1016/j.ijnurstu.2021.103973

[74] Löbel, L. M. & Jacobsen, J. Waiting for kin: A longitudinal study of family reunification and refugee mental health in Germany. *J. Ethnic Migration Stud.* **47** (13), 2916–2937. 10.1080/1369183X.2021.1884538 (2021).

[75] Rudman, L. A., Greenwald, A. G. & McGhee, D. E. Implicit Self-Concept and Evaluative Implicit Gender Stereotypes: Self and Ingroup Share Desirable Traits. *Pers. Soc. Psychol. Bull.* **27** (9), 1164–1178. 10.1177/0146167201279009 (2001).

[76] Rudman, L. A. Self-promotion as a risk factor for women: The osts and benefits of counterstereotypical impression management. *Jour- nal Personality Social Psychol.* **74**, 629–645 (1998). 10.1037//0022-3514.74.3.629 9523410

[77] Henderson, A., Jewell, T., Huang, X. & Simpson, A. Personal trauma history and secondary traumatic stress in mental health professionals: A systematic review. *J. Psychiatr. Ment. Health Nurs.* **32** (1), 13–30. 10.1111/jpm.13082 (2025). 38972012 10.1111/jpm.13082 PMC11704991

[78] Demerouti, E., Bakker, A. B., Nachreiner, F. & Schaufeli, W. B. A model of burnout and life satisfaction amongst nurses. *J. Adv. Nurs.* **32** (2), 454–464. 10.1046/j.1365-2648.2000.01496.x (2000). 10964195 10.1046/j.1365-2648.2000.01496.x

[79] Demerouti, E. & Nachreiner, F. Zum Arbeitsanforderungen-Arbeitsressourcen-Modell von Burnout und Arbeitsengagement – Stand der Forschung. *Z. für Arbeitswissenschaft*. **73** (2), 119–130. 10.1007/s41449-018-0100-4 (2019).

[80] Kovacheva, V. & Grewe, M. *MIgrant workers in the German healthcare sector*. (2015).

[81] UNHCR-UNESCO. *What a waste—Ensure that migrants and refugees’ qualifications and prior learning are recognized*. (2018). https://www.unhcr.org/sites/default/files/legacy-pdf/5c3c6f1f14.pdf

[82] GIZ, & Bundesagentur für Arbeit. *Nachhaltig ausgerichtete Gewinnung von Pflegekräften (Triple Win)*. (2021). https://www.giz.de/de/weltweit/41533.html

[83] Patuzzi, L. & Schröder, S. *Denkfabrik für transnationale Skills Partnerships: Abschlussbericht*. (2024). 10.11586/2024067

[84] Can, E. et al. Foreign Healthcare Professionals in Germany: A Questionnaire Survey Evaluating Discrimination Experiences and Equal Treatment at Two Large University Hospitals. *Healthcare* **10** (12), 2339. 10.3390/healthcare10122339 (2022). 36553863 10.3390/healthcare10122339 PMC9777572

[85] Bundesminesterium für Gesundheit. *Vielfalt stärken – Gesundheit fördern*. (2023). https://www.bundesgesundheitsministerium.de/themen/internationale-gesundheitspolitik/migration-und-integration.html

[86] Expert Council on Integration and Migration. *Jahresgutachten—Kontinuität oder Paradigmenwechsel? Die Integrations- und Migrationspolitik der letzten Jahre*. (2024). https://www.svr-migration.de/wp-content/uploads/2024/06/Jahresgutachten-2024-Barrierefrei.pdf

[87] Halady, E. & Cook-Cottone, C. Mindful self-care, coping, and meaning in life: An examination of the professional quality of life and well-being among individuals who support and provide services to refugees. *Psychol. Trauma: Theory Res. Pract. Policy*. **15** (Suppl 2), S465–S473. 10.1037/tra0001502 (2023). 10.1037/tra0001502 38885429

[88] Wang, X. & Cheng, Z. Cross-Sectional Studies. *Chest* **158** (1), S65–S71. 10.1016/j.chest.2020.03.012 (2020). 32658654 10.1016/j.chest.2020.03.012

[89] Althubaiti, A. Information bias in health research: Definition, pitfalls, and adjustment methods. *J. Multidisciplinary Healthc.* 211. 10.2147/JMDH.S104807 (2016). 10.2147/JMDH.S104807 PMC4862344 27217764

[90] Maxwell, S. E. & Delaney, H. D. *Designing experiments and analyzing data: A model comparison perspective* (2nd ed (Online-Ausg.)). Lawrence Erlbaum Associates. (2004).

[91] Polit, D. F. & Beck, C. T. Generalization in quantitative and qualitative research: Myths and strategies. *Int. J. Nurs. Stud.* **47** (11), 1451–1458. 10.1016/j.ijnurstu.2010.06.004 (2010). 20598692 10.1016/j.ijnurstu.2010.06.004

